# Sex Differences in Response to Metformin in a Longitudinal Study in Mice

**DOI:** 10.1093/geroni/igaa057.1673

**Published:** 2020-12-16

**Authors:** Camila Vieira Ligo Teixeira, Irene Alfaras, Simonetta Camadola, Nathan Price, Michel Bernier, Rafael de Cabo

**Affiliations:** 1 National Institute on Aging,, Baltimore, Maryland, United States; 2 University of Pittsburgh, Pittsburgh, Pennsylvania, United States; 3 Yale University, New Haven, Connecticut, United States

## Abstract

Sexual dimorphisms have been recognized in most aspects of human health and disease, and there is now clear evidence for more pronounced differences in response to pharmacological interventions of aging. Here, male and female C57BL/6J mice at six months of age were administered 0.5% metformin in their diet every-other-week (EOW) for the remainder of their lives. The intervention was well tolerated and did not result in lifespan extension. Male mice treated with metformin EOW lost weight and augmented lean-to-fat ratio. In contrast, EOW females were refractory to changes in body weight and body composition compared to controls. The intervention did not influence non-fasted plasma glucose levels, while causing an increase in lactate in mice of both sexes. Indirect calorimetry was performed to measure energy expenditure in EOW mice that were either ON or OFF metformin during testing. Focusing on the respiratory exchange ratio (RER), males, but not females, preferentially utilized carbohydrates (RER ~0.9-1.0) when OFF metformin, switching to lipids (RER ~0.8-0.9) when ON metformin. This resulted in significant differences compared to controls in both periods (OFF and ON). RER of EOW females was different from controls during OFF metformin (RER ~0.8-0.9), while exhibiting significantly lower RER when ON metformin (RER ~0.7-0.8). These results clearly point at a strong dimorphism in the action of metformin in mice, including its role in metabolic homeostasis and overall health span. A better understanding of how sex influences the health and prolongevity benefits of metformin will benefit our understanding of the longterm clinical implications.

